# The Cardioprotective PKA-Mediated Hsp20 Phosphorylation Modulates Protein Associations Regulating Cytoskeletal Dynamics

**DOI:** 10.3390/ijms21249572

**Published:** 2020-12-16

**Authors:** Elizabeth Vafiadaki, Demetrios A. Arvanitis, Aristides G. Eliopoulos, Evangelia G. Kranias, Despina Sanoudou

**Affiliations:** 1 Molecular Biology Division, Biomedical Research Foundation of the Academy of Athens, 115 27 Athens, Greece; lvafiadaki@bioacademy.gr (E.V.); arvanitd@bioacademy.gr (D.A.A.); eliopag@med.uoa.gr (A.G.E.); kraniaeg@ucmail.uc.edu (E.G.K.); 2 Department of Biology, School of Medicine, National and Kapodistrian University of Athens, 115 27 Athens, Greece; 3 Center for New Biotechnologies and Precision Medicine, Medical School, National and Kapodistrian University of Athens, 115 27 Athens, Greece; 4 Department of Pharmacology and Systems Physiology, University of Cincinnati, College of Medicine, Cincinnati, OH 45267-0575, USA; 5 Clinical Genomics and Pharmacogenomics Unit, 4th Department of Internal Medicine, Medical School, National and Kapodistrian University of Athens, 115 27 Athens, Greece

**Keywords:** Hsp20 phosphorylation, 14-3-3, actin cytoskeleton, cardiac muscle, cardiomyopathy, heart failure

## Abstract

The cytoskeleton has a primary role in cardiomyocyte function, including the response to mechanical stimuli and injury. The small heat shock protein 20 (Hsp20) conveys protective effects in cardiac muscle that are linked to serine-16 (Ser16) Hsp20 phosphorylation by stress-induced PKA, but the link between Hsp20 and the cytoskeleton remains poorly understood. Herein, we demonstrate a physical and functional interaction of Hsp20 with the cytoskeletal protein 14-3-3. We show that, upon phosphorylation at Ser16, Hsp20 translocates from the cytosol to the cytoskeleton where it binds to 14-3-3. This leads to dissociation of 14-3-3 from the F-actin depolymerization regulator cofilin-2 (CFL2) and enhanced F-actin depolymerization. Importantly, we demonstrate that the P20L Hsp20 mutation associated with dilated cardiomyopathy exhibits reduced physical interaction with 14-3-3 due to diminished Ser16 phosphorylation, with subsequent failure to translocate to the cytoskeleton and inability to disassemble the 14-3-3/CFL2 complex. The topological sequestration of Hsp20 P20L ultimately results in impaired regulation of F-actin dynamics, an effect implicated in loss of cytoskeletal integrity and amelioration of the cardioprotective functions of Hsp20. These findings underscore the significance of Hsp20 phosphorylation in the regulation of actin cytoskeleton dynamics, with important implications in cardiac muscle physiology and pathophysiology.

## 1. Introduction

The small heat shock protein 20 (Hsp20, also referred to as HspB6) is a 17 kDa molecular chaperone with a central role in cardiac function [1,2]. Hsp20 is transiently upregulated in the heart as a compensatory protective response to stress factors, such as exercise training, chronic β-adrenergic stimulation and ischemic injury, as well as in human and experimental heart failure [1,3,4,5,6]. This cardioprotective role of Hsp20 has been shown to involve attenuation of apoptosis, reduced infarction and improved recovery of cardiac function [5,6,7,8,9].

Importantly, the cardioprotective effects of Hsp20 are linked to Ser16 phosphorylation by cAMP-dependent protein kinase (PKA) [10,11]. This has been demonstrated by adenoviral overexpression of Hsp20 in isolated cardiomyocytes, as well as animal model studies expressing non-phosphorylatable Hsp20 (S16A) or phosphomimetic Hsp20 (S16D) following β adrenergic stimulation [5,6]. In particular, blockade of Hsp20-Ser16 phosphorylation in vivo was found to exacerbate cardiac ischemia/reperfusion injury and increase cardiomyocyte apoptosis. Conversely, the constitutively phosphorylated Hsp20-S16D conferred full protection from cardiomyocyte apoptosis via inhibition of caspase 3 [5]. These findings indicate the critical requirement of Ser16 phosphorylation in exerting the cardioprotective properties of Hsp20, with potential therapeutic applications in cardiac disease.

In addition to evidence from experimental models, the identification of Hsp20 mutations in dilated cardiomyopathy (DCM) patients has further supported the functional significance of Hsp20 in cardiac muscle [12,13]. The first Hsp20 mutation to be reported was a single base substitution that changes proline into leucine at amino acid position 20 (P20L). Adenovirus-mediated overexpression of Hsp20-P20L in isolated cardiomyocytes demonstrated that the mutation abrogates the cardioprotective properties of Hsp20. This was associated with diminished phosphorylation at Ser16 upon simulated ischemia/reperfusion or isoproterenol treatment in cardiac myocytes. Based on these findings, it was proposed that the P20L mutation results in impaired ability of cardiomyocytes to handle cellular stress. The critical role of Hsp20 in cardiac physiology and pathophysiology was further highlighted by the identification of a second mutation (S10F) in DCM patients [12]. Cardiac overexpression of Hsp20-S10F was associated with cardiac dysfunction, heart failure and reduced survival in male mice and peripatrum cardiomyopathy in female mice [12,14].

Mechanistically, the protective properties of Hsp20 can be attributed to its association with various binding partners regulating cell survival or death, phosphorylation enzymes or subunits, as well as protein phosphatases or phosphodiesterases [7,10,15,16,17,18,19]. Previous studies in non-muscle tissues have reported association of Hsp20 with the cytoskeletal protein 14-3-3, an adaptor molecule interacting with multiple proteins with diverse functions [20,21,22]. Among the interacting partners of 14-3-3 is cofilin-2 (CFL2), a protein that modulates actin dynamics in striated muscle [21,23]. Moreover, Hsp20 has been involved in cytoskeletal regulation through interaction with key cytoskeletal proteins such as actin and α-actinin [5,24]. However, the physical interactions of Hsp20 with cytoskeletal complexes and their functional implications in cardiac muscle remain unknown.

Data presented herein underscore a significant role for the cardioprotective Hsp20-Ser16 phosphorylation in modulating the subcellular distribution and interaction of Hsp20 with 14-3-3, leading to dissociation of CFL2 from 14-3-3 and enhanced CFL2-mediated F-actin depolymerization. Conversely, the human Hsp20-P20L mutation which is causally linked to impaired cardiac function displays reduced phosphorylation and diminished physical interaction with 14-3-3 leading to aberrant regulation of CFL2-mediated F-actin depolymerization. Collectively, these findings link PKA-mediated Hsp20 phosphorylation to cytoskeletal changes pertinent to cardiac muscle physiology and pathophysiology.

## 2. Results

### 2.1. Identification of Hsp20/14-3-3 and 14-3-3/CFL2 Protein Complexes in Cardiac Muscle

**Figure 1:**
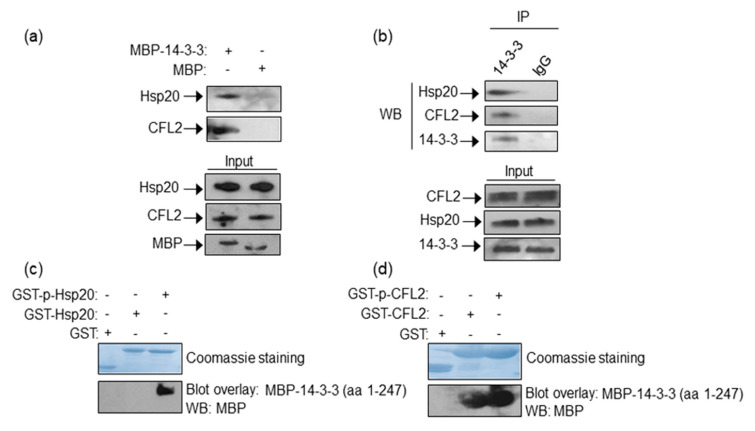
Identification of Hsp20/14-3-3 and 14-3-3/CFL2 protein complexes in cardiac muscle. (**a**) MBP-14-3-3 pull down assays determine its interactions with Hsp20 and CFL2 in cardiac homogenates. (**b**) Immunoprecipitation assays using 14-3-3 or IgG control antibodies confirm the association of 14-3-3/Hsp20 and 14-3-3/CFL2 endogenously in cardiac muscle extracts. (**c**,**d**) Coomassie staining showing purified GST-Hsp20 and GST-CFL2 recombinant proteins. Hsp20/14-3-3 and 14-3-3/CFL2 interactions are direct, as shown by blot overlay assays. Specifically, 14-3-3 interacts with (**c**) phosphorylated Hsp20 and (**d**) both phosphorylated and non-phosphorylated CFL2, as determined by two independent experiments. No unspecific binding to MBP has been observed in control experiments (Figure S1).

To determine whether 14-3-3 interacts with Hsp20 and CFL2 in the heart, we performed pull down assays using recombinant MBP-14-3-3 or MBP proteins and mouse cardiac protein extracts. Western blot analysis showed that in vitro expressed 14-3-3 binds Hsp20 and CFL2 in cardiac muscle (Figure 1a). To determine if these interactions may also occur between endogenously expressed proteins, we examined the presence of Hsp20 and CFL2 in anti-14-3-3 immunoprecipitates from cardiac muscle lysates. Hsp20 and CFL2 were found to co-precipitate with 14-3-3 (Figure 1b), thereby confirming the existence of these interactions endogenously in cardiac muscle.

As Hsp20 function is affected by phosphorylation at Ser16 by PKA [10,25], we examined the impact of phosphorylation in the identified 14-3-3 protein interactions. Towards this, we performed blot overlay assays using GST-Hsp20 or GST-CFL2 recombinant proteins that had been previously phosphorylated in vitro, or left untreated for control purposes (Figure 1c). These in vitro assays determined that 14-3-3 interacts preferentially with phosphorylated-Hsp20, whereas GST-CFL2 associates with 14-3-3 irrespective of its phosphorylation status (Figure 1d). The existence of low levels of phosphorylated Hsp20 in cardiac extracts (as shown in Figure 3c) explains the ability to detect the Hsp20 and 14-3-3 interaction endogenously in cardiac extracts by pull down and immunoprecipitation assays (Figure 1a,b).

We conclude that CFL2 and Hsp20 may bind directly and independently of each other to 14-3-3 in cardiac muscle. Moreover, binding of Hsp20 to 14-3-3 is modulated by PKA-mediated phosphorylation, indicating the presence of a regulatory protein complex with a putative functional significance during β-adrenergic stimulation.

### 2.2. Mapping of the Minimal Regions of Hsp20 and CFL2 Responsible for Binding to 14-3-3

**Figure 2:**
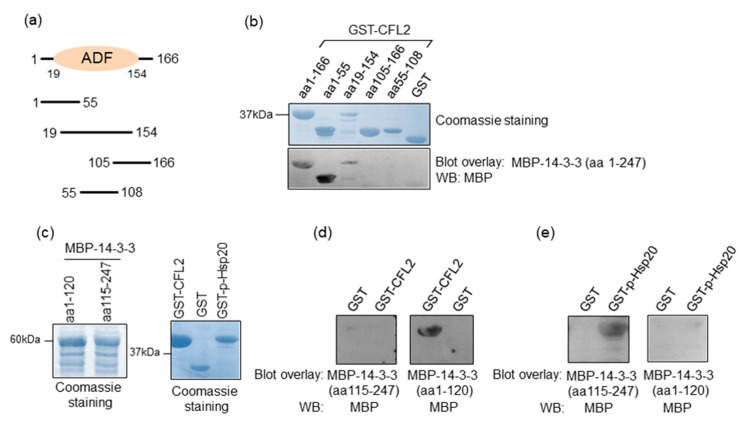
Minimal binding regions of interactions. (**a**) Diagrammatic representation of the various CFL2 deletion constructs. ADF, actin-depolymerizing factor. (**b**) Coomassie blue stained gel showing purified recombinant GST and GST-CFL2 deletion proteins. Blot overlay assays using GST-CFL2 deletion constructs and MBP-14-3-3 determine that 14-3-3 binds to N-terminal region of CFL2 that includes amino acids 1-55. (**c**) Coomassie blue stained gel showing purified MBP-14-3-3 deletion proteins as well as GST-CFL2, GST and GST-p-Hsp20 recombinant proteins. (**d**,**e**) Blot overlay assays using MBP-14-3-3 N- or C-terminal deletion constructs determine the regions of 14-3-3 that bind to Ser16 phosphorylated-Hsp20 and CFL2. No unspecific binding to MBP was observed in control experiments (Figure S1).

The observation that 14-3-3 binds directly to Hsp20 and CFL2 in cardiac muscle prompted us to investigate the minimal regions of CFL2 and Hsp20 involved in 14-3-3 binding. For this purpose, we first generated recombinant proteins from various deletion constructs of CFL2 (Figure 2a,b). Blot overlay assays with 14-3-3 showed that the N-terminal fragment of CFL2, containing amino acids 1-55, is required for binding to 14-3-3 (Figure 2b).

We next mapped the minimal region of 14-3-3 that is required for association with CFL2 and Hsp20 by generating N- and C-terminal deletion constructs of 14-3-3 (Figure 2c). Blot overlay assays with GST-CFL2 demonstrated that the N-terminal region of 14-3-3, containing amino acids 1–120, is required for binding to CFL2 (Figure 2d). In contrast, the binding of 14-3-3 to phosphorylated Hsp20 requires the C-terminal fragment of 14-3-3 encompassing amino acids 115–247 (Figure 2e). We conclude that different regions of 14-3-3 are involved in interactions with Hsp20 and CFL2.

### 2.3. Hsp20 Competes with CFL2 for Binding to 14-3-3

**Figure 3:**
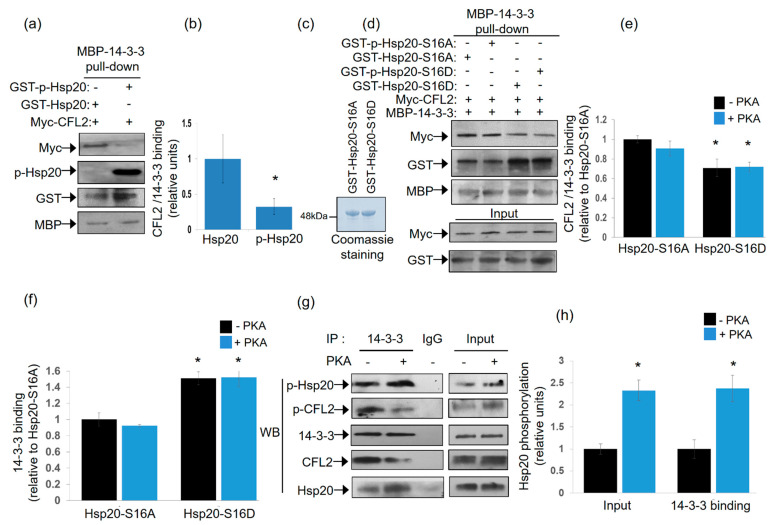
Phosphorylated Hsp20 displaces CFL2 from 14-3-3 protein complex. (**a**) Pull down assays using MBP-14-3-3 recombinant protein and lysates from HEK 293 cell transfected with myc-CFL2. Binding of CFL2 to 14-3-3 was examined in the presence of GST-Hsp20 or Ser16-phosphorylated GST-Hsp20. (**b**) Quantification of CFL2/14-3-3 binding in the presence of GST-Hsp20 or Ser16-phosphorylated GST-Hsp20 (* *p* < 0.05, *t* test, two-tailed; *n* = 3). Data are the mean ± SD. (**c**) Coomassie blue stained gel showing purified recombinant GST-Hsp20-S16A and GST-Hsp20-S16D recombinant proteins. (**d**) Pull down assays using MBP-14-3-3 recombinant protein and lysates from HEK 293 cell transfected with myc-CFL2. Binding of CFL2 to 14-3-3 was examined in the presence of GST-Hsp20-S16A, GST-Hsp20-S16D, or phosphorylated GST-Hsp20-S16A and phosphorylated GST-Hsp20-S16D. (**e**) Quantification of CFL2/14-3-3 binding in the presence of GST-Hsp20-S16A or GST-Hsp20-S16D (* *p* < 0.05 vs. Hsp20-S16A -PKA, *t* test, two-tailed; *n* = 3). Data are the mean ± SD. (**f**) Quantification of 14-3-3 binding to GST-Hsp20-S16A or GST-Hsp20-S16D (* *p* < 0.05 vs. Hsp20-S16A-PKA, *t* test, two-tailed; *n* = 3). Data are the mean ± SD. (**g**) Immunoprecipitation assays in PKA-phosphorylated or non-phosphorylated cardiac extracts confirmed the enhanced binding of Ser-16 phosphorylated Hsp20 to 14-3-3, along with associated reduction in the phosphorylated CFL2/14-3-3 interaction. (**h**) Quantification of the impact of PKA treatment on the levels of Hsp20 phosphorylation in cardiac extracts (input) and 14-3-3 interaction (* *p* < 0.05, *t* test, two-tailed; *n* = 3). Data are the mean ± SD.

As both Hsp20 and CFL2 interact with 14-3-3, albeit at different regions of the protein, we proceeded to assess whether protein binding can occur simultaneously or in a mutually exclusive manner. To this end, we performed pull down assays using MBP-14-3-3 recombinant protein and lysates from HEK 293 cells transfected with myc-tagged-CFL2, in the presence of phosphorylated or non-phosphorylated recombinant GST-Hsp20 protein. Immunoblot analysis of MBP-14-3-3 precipitates showed reduced binding of CFL2 in the presence of phosphorylated, compared to non-phosphorylated, Hsp20 (Figure 3a,b, * *p* < 0.05, *t* test, two-tailed *n* = 3). This was associated with increased binding of phosphorylated GST-Hsp20 to MBP-14-3-3, in agreement to the findings from blot overlay and immunoprecipitation assays (Figure 1c). To further evaluate the significance of Hsp20 phosphorylation on this, we assessed the effect of GST-Hsp20-S16A and GST-Hsp20-S16D phospho-mutant proteins (Figure 3c). GST-Hsp20-S16A did not appear to affect myc-CFL2 interaction to MBP-14-3-3 upon phosphorylation (Figure 3d,e). This could be attributed to the fact that Hsp20-S16A is a non-phosphorylatable protein at Ser-16. On the other hand, GST-Hsp20-S16D exhibited increased binding to MBP-14-3-3 (Figure 3d,f), with a concomitant overall decrease of myc-CFL2 association to MBP-14-3-3 (Figure 3d,e). No major difference is seen between GST-p-Hsp20-S16D and GST-Hsp20-S16D samples since Hsp20-S16D is a phosphomimetic protein at Ser-16 and is consequently constitutively phosphorylated. Similar findings on the impact of phosphorylation on the association of Hsp20 with 14-3-3 were obtained by immunoprecipitation of endogenous protein complexes in PKA-phosphorylated and non-phosphorylated mouse cardiac protein extracts. As shown in Figure 3g,h, the association of 14-3-3 with Hsp20 increased in PKA-phosphorylated extracts, whereas interaction with CFL2 decreased. These findings reveal the competitive nature of the CFL2 and Hsp20 association with 14-3-3 and the critical role of Hsp20 phosphorylation by PKA in these interactions.

### 2.4. Isoproterenol-Induced Phosphorylation of Hsp20 Changes Its Subcellular Distribution by Promoting Its Interaction with 14-3-3

**Figure 4:**
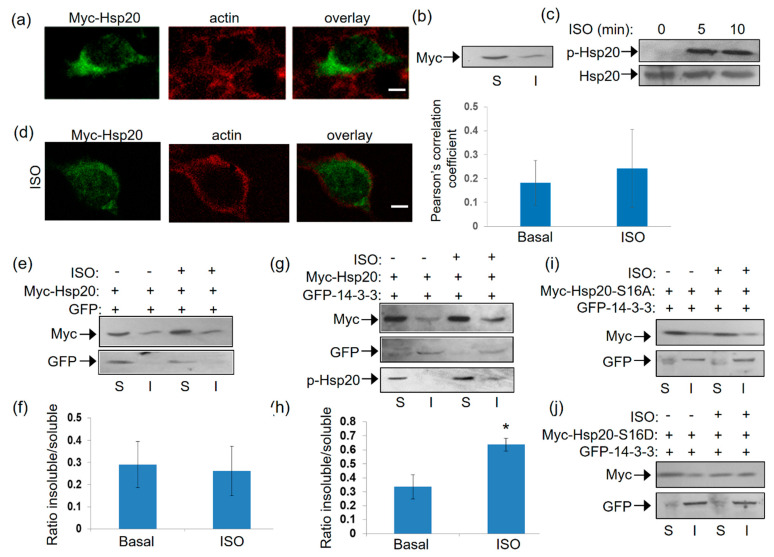
Localization of Hsp20 protein in HEK 293 cells and redistribution in the presence of 14-3-3. (**a**) Immunofluorescence analysis of HEK 293 cells transfected with myc-tagged-Hsp20 and examination of its localization with the cytoskeletal marker, actin. Scale bar 5 μm. (**b**) Fractionation study of myc-Hsp20 and examination of its localization in cytosolic soluble (S) and cytoskeletal insoluble (I) cell fractions. (**c**) 1 μM ISO treatment results in efficient Ser16 phosphorylation of myc-Hsp20. (**d**) Immunofluorescence analysis of HEK 293 cells transfected with myc-tagged-Hsp20 and examination of its localization with the cytoskeletal actin following ISO treatment. Calculation of Pearson’s correlation coefficient determined no significant alteration in the subcellular distribution of myc-Hsp20 following ISO treatment. Data are the mean ± SD; *n* = 15 cells from each group. Scale bar 5 μm. (**e**) Fractionation of ISO treated HEK 293 cells transfected with myc-Hsp20 and GFP vector or (**g**) GFP-14-3-3 construct and determination of myc-Hsp20 subcellular location. (**f**,**h**) Quantification of myc-Hsp20 distribution in the presence of GFP or GFP-14-3-3 (* *p* < 0.05, *t* test, two-tailed; *n* = 3). Data are the mean ± SD. (**i**) Fractionation of ISO treated HEK 293 cells transfected with myc-Hsp20-S16A and GFP-14-3-3 or (**j**) myc-Hsp20-S16D and GFP-14-3-3 constructs and determination of their subcellular location.

Having established the association of 14-3-3 with Hsp20 in vitro and the influence of Hsp20 phosphorylation thereof, we next examined the nature of this interaction under conditions of endogenous activation of PKA ensued by isoproterenol (ISO) treatment. ISO is an agonist of the β2-adrenergic receptor (β2AR) which elevates cAMP and thus, PKA activity. To this end, we first transfected HEK 293 cells with a myc-tagged Hsp20 expression vector and found by immunofluorescence that Hsp20 preferentially localizes to the cytosol (Figure 4a). This observation was reproduced using crude fractionation assays in detergent-soluble (cytosolic) and detergent-insoluble (cytoskeletal) fractions (Figure 4b), consistent with previous findings in cardiomyocytes [5].

Given that Hsp20 phosphorylation affects its interaction with 14-3-3 (Figure 1), we proceeded to assess the levels and subcellular distribution of phosphorylated Hsp20 following treatment with ISO. ISO induced a rapid (within 5 min) phosphorylation of Hsp20 at Ser16 which was maintained at later time points (Figure 4c). ISO treatment did not result in major changes in Hsp20 localization, as observed by immunofluorescence analysis (Figure 4d). To determine the effect of phosphorylation on 14-3-3/Hsp20 localization, we used more detailed biochemical assays and performed crude fractionation assays on myc-tagged Hsp20-transfected HEK 293 cells in the presence of GFP-14-3-3 or GFP as control. In GFP-transfected cells, ISO treatment did not affect Hsp20 subcellular distribution to soluble versus insoluble fraction (Figure 4e,f). However, ISO treatment of cells co-transfected with GFP-14-3-3 led to an increase in the amount of myc-Hsp20 in the cytoskeletal fraction where 14-3-3 predominantly resides (Figure 4g,h), * *p* < 0.05, *t* test, two-tailed *n* = 3). Moreover, the cytoskeletal fraction contained phosphorylated Hsp20 (Figure 4g) suggesting that its redistribution occurs in phosphorylation-dependent manner. This is clearly demonstrated by analysis of the phospho-mutant constructs myc-Hsp20-S16A and myc-Hsp20-S16D which served as negative and positive controls in our study. Indeed, ISO treatment did not enhance the distribution of myc-HSp20-S16A to the insoluble fraction, while myc-Hsp20-S16D exhibited a more equal distribution in both soluble and insoluble fractions (Figure 4i,j). These findings underscore the role of Hsp20 phosphorylation in regulating its interaction with 14-3-3 and thus, its subcellular localization.

### 2.5. The Human Phosphorylation-Impairing Hsp20-P20L Mutation Displays Reduced 14-3-3 Interaction

**Figure 5:**
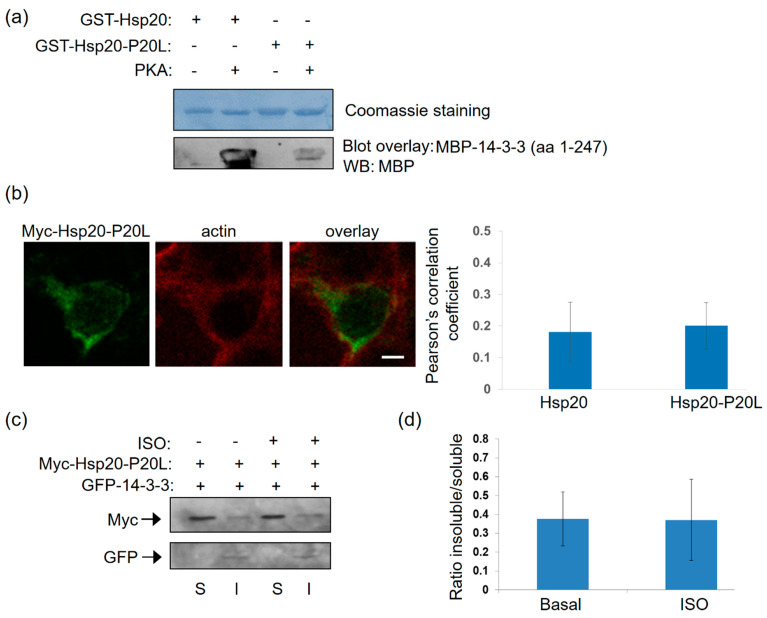
The human P20L mutation in Hsp20 inhibits its association with 14-3-3. (**a**) Coomassie staining of GST-Hsp20 recombinant proteins. Blot overlay assays using GST-Hsp20-WT or P20L and MBP-14-3-3 determine diminished binding of PKA-treated P20L mutant protein to 14-3-3. No unspecific binding to MBP was observed in control experiments (Figure S1). (**b**) Immunofluorescence analysis of HEK 293 cells transfected with myc-Hsp20-P20L and examination of its localization with the cytoskeletal marker, actin. Calculation of Pearson’s correlation coefficient determined no significant alteration in the subcellular distribution of myc-Hsp20-P20L, when compared to myc-Hsp20. Data are the mean ± SD; *n* = 15 cells from each group. Scale bar 5 μm. (**c**) Fractionation of 1 μM ISO treated HEK 293 cells transfected with myc-Hsp20-P20L and GFP-14-3-3 determines no significant alteration of myc-Hsp20-P20L subcellular location. (**d**) Quantification of myc-Hsp20-P20L distribution in the presence GFP- 14-3-3. Data are the mean ± SD; *n* = 3.

The P20L mutation of Hsp20 found in patients with dilated cardiomyopathy, is linked to reduced Hsp20 phosphorylation and abrogation of its cardioprotective effects [13]. As Hsp20 phosphorylation impacts on its interaction with 14-3-3, we reasoned that Hsp20-P20L may display reduced binding to 14-3-3. In line with this prediction, blot overlay assays demonstrated that recombinant Hsp20-P20L has significantly reduced capacity to bind to 14-3-3 upon PKA treatment, when compared to the WT Hsp20 (Figure 5a).

Subcellular analysis of Hsp20-P20L-transfected HEK 293 cells demonstrated no major alterations in the localization of the mutated versus WT protein (Figure 4a and Figure 5b). This was confirmed by cell fractionation experiments, which demonstrated primarily cytosolic localization of Hsp20-P20L, similar to the WT protein. However, unlike WT Hsp20 (Figure 4g), P20L could not successfully translocate to the cytoskeletal fraction containing GFP-14-3-3 upon ISO treatment (Figure 5c,d, *n* = 3). We conclude that the naturally occurring Hsp20-P20L mutation displays diminished 14-3-3 interaction and mobilization to the cytoskeletal fraction under stress conditions, such as β-adrenergic stimulation.

### 2.6. Hsp20-P20L Fails to Compete with CFL2 for Binding to 14-3-3

**Figure 6:**
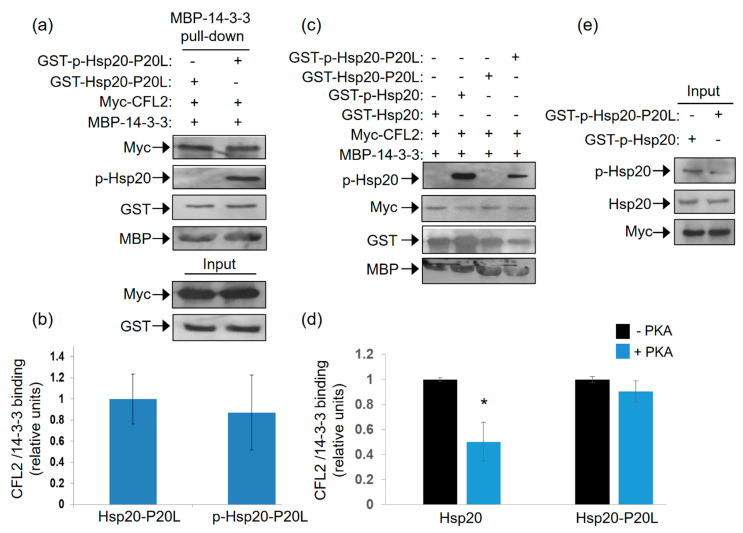
Ser16- Hsp20-P20L fails to displace CFL2 from 14-3-3 protein complex. (**a**) Pull down assays using MBP-14-3-3 recombinant protein and lysates from HEK 293 cells transfected with myc-CFL2. Binding of CFL2 to 14-3-3 was examined in the presence of GST-Hsp20-P20L or Ser16-phosphorylated GST-Hsp20-P20L. (**b**) Quantification of CFL2/14-3-3 binding in the presence of GST-Hsp20-P20L or Ser16-phosphorylated GST-Hsp20-P20L. Data are the mean ± SD; *n* = 3. (**c**) Representative pull down assay showing the diminished effect of Hsp20-P20L in competing with CFL2 for 14-3-3 binding, when compared to WT Hsp20. (**d**) Quantification of CFL2/14-3-3 binding in the presence of GST-Hsp20 or GST-Hsp20-P20L (* *p* < 0.05 vs. Hsp20-PKA, *t* test, two-tailed; *n* = 3). Data are the mean ± SD; *n* = 3. (**e**) Reduced Ser16 phosphorylation of Hsp20-P20L mutant, in comparison to Hsp20-WT protein.

As the P20L mutation diminishes the association of Ser16 phosphorylated-Hsp20 with 14-3-3 (Figure 5), and given the competitive nature of Hsp20 and CFL2 binding to 14-3-3 (Figure 3), we hypothesized that the Hsp20-P20L mutation may not impair the 14-3-3 interaction with CFL2. To address this hypothesis, we tested the capacity of recombinant MBP-14-3-3 protein to bind myc-CFL2 expressed in HEK 293 cells in the presence of recombinant phosphorylated versus non-phosphorylated GST-Hsp20-P20L. The results of the pull down assays showed that GST-Hsp20-P20L treated with PKA did not reduce the interaction of 14-3-3 with CFL2 (Figure 6a,b). This was associated with significantly reduced phosphorylated Hsp20-P20L protein binding to MBP-14-3-3 in the pull down sample (Figure 6c,d). This is also evident by immunoblot analysis with GST antibody, showing that phosphorylation of WT Hsp20 resulted in enhanced binding to MBP-14-3-3, however, this effect was not observed for GST-Hsp20-P20L (Figure 6c). As a consequence, in the presence of phosphorylated GST-Hsp20-P20L there was no significant reduction in 14-3-3 binding to CFL2, in contrast to WT Hsp20 (Figure 6c,d). These findings indicate inability of Hsp20-P20L to compete with CFL2 for 14-3-3 binding, compared to WT Hsp20 (Figure 6c,d). In line with the observation that Hsp20 phosphorylation affects its interaction with 14-3-3 (Figure 1), Hsp20-P20L displayed reduced phosphorylation at residue Ser16 (Figure 6e). This finding is consistent with our observation of reduced Hsp20-P20L binding to 14-3-3 following β-adrenergic stimulation.

Collectively, these data suggest that the Hsp20-P20L mutation not only affects the direct interaction of this protein with 14-3-3, but it also impairs the regulation of the CFL2/14-3-3 interaction.

### 2.7. Hsp20 Phosphorylation Regulates CFL2-Mediated Actin Depolymerization

**Figure 7:**
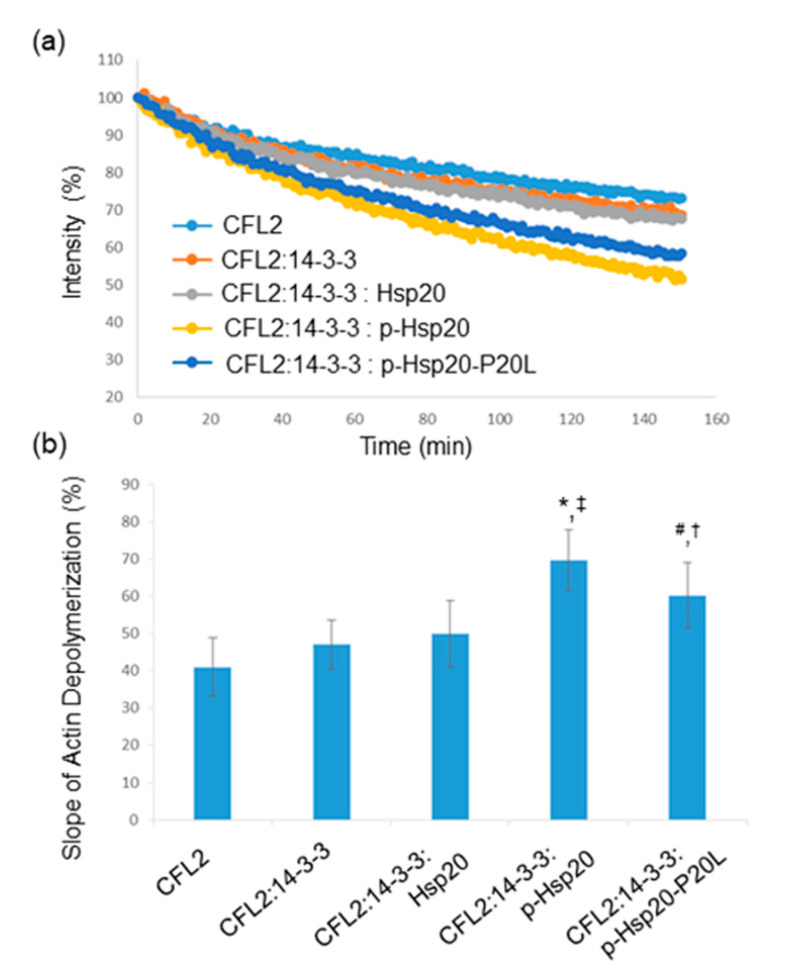
Hsp20 phosphorylation enhances CFL2 mediated actin depolymerization. (**a**) In vitro depolymerization assays were performed in the presence of GST-CFL2, MBP-14-3-3 and GST-Hsp20 recombinant proteins. Phosphorylated Hsp20 enhanced depolymerization of F-actin by CFL2, while this effect was reduced in the presence of phosphorylated Hsp20-P20L mutant protein. (**b**) Calculation of the slope of F-actin depolymerization of the different protein combinations. (* *p* < 0.05 vs. CFL2; ^#^ *p* < 0.05 vs. CFL2:14-3-3:phospho-Hsp20; ^†^ *p* < 0.05 vs. CFL2:14-3-3:Hsp20; ^‡^ *p* < 0.05 vs. CFL2:14-3-3: Hsp20, *t*-test, two-tailed; *n* = 3). Data are the mean ± SD.

CFL2 is a major regulator of actin dynamics and a key player in actin depolymerization [26]. We therefore evaluated whether Hsp20 phosphorylation may regulate CFL2-mediated actin depolymerization. To this end, we performed in vitro pyrene-F-actin depolymerization assays using equivalent amounts of recombinant GST-CFL2, MBP-14-3-3 and PKA-phosphorylated or non-phosphorylated GST-Hsp20 proteins. The results show that addition of 14-3-3, in the presence or absence of Hsp20, did not influence the severing activity of CFL2 (Figure 7a,b). In contrast, the addition of phosphorylated Hsp20 protein in the depolymerization reaction along with 14-3-3 resulted in increased CFL2-mediated actin depolymerization (Figure 7a,b, * *p* < 0.05 vs. CFL2 and ^‡^ *p* < 0.05 vs. CFL2:14-3-3:Hsp20, *t*-test, two-tailed, *n* = 3). Importantly, compared to Hsp20-WT, the phosphorylated Hsp20-P20L protein displayed a significantly reduced effect on CFL2 activity (Figure 7a,b, ^#^ *p* < 0.05 vs. CFL2:14-3-3:phospho-Hsp20, *t*-test, two-tailed, *n* = 3). However, phosphorylated Hsp20-P20L exhibited significantly enhanced CFL2-mediated actin depolymerization when compared to non-phosphorylated Hsp20 (^†^ *p* < 0.05 vs. CFL2:14-3-3: Hsp20, *t*-test, two-tailed; *n* = 3). Preliminary findings on Hsp20-S16A and Hsp20-S16D indicate that PKA-treatment of Hsp20-S16A had no effect on CFL2 activity, while PKA-treated Hsp20-S16D exhibited enhanced CFL2-mediated F-actin depolymerization, similar to phosphorylated GST-Hsp20 (data not shown). Collectively, these findings reveal an important role of Hsp20 phosphorylation in the regulation of actin dynamics.

## 3. Discussion

**Figure 8:**
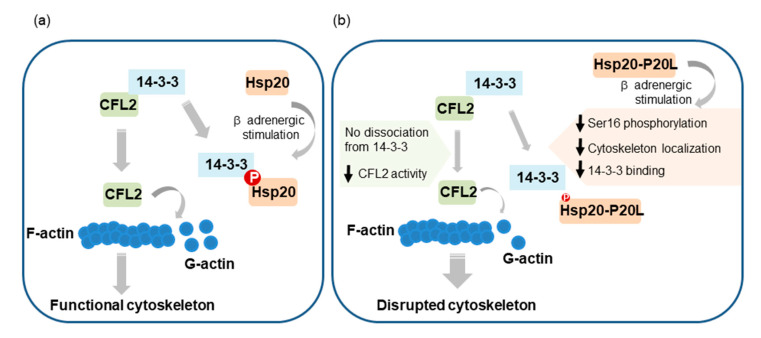
Graphical representation of the proposed contribution of Hsp20 phosphorylation to the regulation of actin cytoskeleton dynamics. (**a**) Hsp20 phosphorylation by PKA during β-adrenergic stimulation leads to Hsp20 subcellular translocation to the cytoskeleton, where it interacts with 14-3-3. For this association to occur, phosphorylated Hsp20 competes with CFL2 for 14-3-3-binding that results in CFL2 dissociation from 14-3-3. This triggers CFL2 activation, leading to enhanced F-actin depolymerization activity and regulation of actin dynamics in order to maintain cytoskeletal integrity and function. (**b**) The human phosphorylation impairing Hsp20-P20L mutated protein exhibits diminished phosphorylation at Ser16 and significantly reduced binding to 14-3-3, with consequent failure to displace CFL2 from 14-3-3. As a result, aberrant regulation of CFL2-mediated F-actin depolymerization. This would lead to cytoskeletal disruption and myofibrillar damage during stress conditions, contributing towards cardiac dysfunction.

β-Adrenergic stimulation of cardiomyocytes, such as under stress conditions, leads to activation of PKA and phosphorylation of key proteins governing Ca^2+^ cycling and contractility. Among them, Hsp20 and its phosphorylation have emerged as important determinants of cardiac muscle function [10,11]. However, the regulatory mechanisms underpinning these events are only partly characterized. Herein, we present evidence that PKA-mediated phosphorylation of Hsp20 regulates its subcellular distribution, with important downstream functional implications pertinent to the cardioprotective role of Hsp20 (Figure 8).

In cardiomyocytes, Hsp20 has been described to exhibit cytosolic localization [27,28], however, following β-adrenergic stimulation, it has been shown to translocate to myofibrils and co-localize with actin [5]. We herein demonstrated that the intracellular redistribution of Hsp20 involves its association with 14-3-3, a cytoskeletal protein that serves as an adaptor molecule regulating target proteins containing specific phosphoserine motifs [29,30,31,32]. Moreover, our findings indicate that this interaction is modulated by PKA-mediated phosphorylation of Hsp20 at Ser16, potentially bringing phosphorylated Hsp20 into proximity with other cytoskeletal proteins and modulating their function. Indeed, treatment of 3T3 cells with a phosphopeptide analog of HSP20 was shown to alter focal adhesion protein localization and regulate actin cytoskeleton [21].

To date, 14-3-3 proteins have been estimated to interact with over 200 proteins [33,34] that are involved in a wide range of biological processes including cell signaling, regulation of cell cycle progression, intracellular trafficking/targeting, cytoskeletal structure and transcription [29,31,35,36]. The effects of 14-3-3 on their targets include: (1) conformational changes; (2) physical occlusion of sequence-specific or structural protein features of its binding partner; or (3) scaffolding by anchoring proteins within close proximity [36,37]. The latter may be of particular relevance to the 14-3-3/Hsp20 complex in the context of cardiac function. This is supported by the fact that upon binding to 14-3-3, Hsp20 translocates to the cytoskeleton where it can interact with α-actinin and actin [5,24]. In addition, Hsp20 has previously been reported to affect cytoskeletal dynamics [21], including F-actin depolymerization in rat aorta [38], although the mechanisms underlying these functions remained undefined. We herein demonstrated that through its binding to 14-3-3, Hsp20 modulates the 14-3-3/CFL2 complex formation, with direct implications on actin-depolymerization. Importantly, we show that the dissociation of 14-3-3 from CFL2 depends on Hsp20 phosphorylation. Thus, upon β-adrenergic stimulation and PKA activation, phosphorylated Hsp20 competes with CFL2 for 14-3-3-binding, resulting in displacement of CFL2 from the 14-3-3 protein complex (Figure 8). Displacement from 14-3-3 is unlikely to be related to CFL2 phosphorylation, since in our competition protein binding assays (shown in Figure 3a) only non-phosphorylated CFL2 was used. Release of CFL2 from 14-3-3 renders it susceptible to phosphatases leading to its dephosphorylation [21]. This ultimately triggers CFL2 activation, leading to enhanced F-actin depolymerization activity.

The identification of the mechanism mediating Hsp20 translocation to the cytoskeleton and modulation of F-actin depolymerization may have important implications in deciphering the involvement of Hsp20 in cardiac disease. Hsp20 has been shown to translocate from the cytosol to myofibrils following cardiac and skeletal muscle ischemia [5,39]. Ischemia is an established trigger of apoptosis [40], which in turn leads to a series of cytoskeletal modifications including cytoskeletal disruption due to cleavage of actin and alterations in actin dynamics [41,42,43]. The stress-induced, phosphorylation-dependent translocation of Hsp20 to myofibrils protects against apoptosis [5,39] and promotes cytoskeletal integrity. Mice overexpressing Hsp20 exhibit significantly reduced myofibril damage and myocardial infarct size following ischemia–reperfusion injury [7]. Given that cytoskeletal disruption plays a crucial role in the pathogenesis of myocardial ischemic injury [44,45], our findings underscore the potential of Hsp20 phosphorylation in promoting cardiomyocyte cytoskeleton integrity, protection from apoptosis and prevention of myocardial dysfunction. In line with these findings, the upregulation of other Hsp proteins, such as Hsp27, has been shown to serve as an adaptive mechanism towards stabilization and protection of the actin cytoskeleton to prevent its disruption [46,47,48,49,50,51,52].

The relevance of this regulatory mechanism to heart disease pathogenesis is further underscored by the molecular and cellular implications of the phosphorylation impairing Hsp20-P20L mutation that has been observed in dilated cardiomyopathy patients [13,53]. At the cellular level, Hsp20-P20L displays reduced cardioprotective properties under stress conditions [13], while at the molecular level, these effects have been attributed to the reduced phosphorylation of Hsp20-P20L at Ser16 [13].

Our findings demonstrate that the impaired ability of mutant Hsp20 to become phosphorylated at Ser16, leads to significantly reduced binding to 14-3-3, and aberrant regulation of CFL2-mediated F-actin depolymerization (Figure 8), possibly contributing towards cardiac pathogenesis under stress conditions. In line with this notion, lack of CFL2 regulation, as observed in CFL2-deficient mouse models or human mutations of CFL2, results in progressive sarcomeric disruption and actin accumulation due to reduced depolymerization of actin filaments that lead to myopathy [54,55,56].

In conclusion, we herein describe a mechanism through which β-adrenergic stimulation modulates Hsp20 subcellular localization and function in cardiomyocytes. We show that the PKA-mediated phosphorylation of Hsp20 promotes its translocation to the cytoskeleton where it modulates the interaction of 14-3-3 with CFL2 and consequently impacts F-actin depolymerization. Through this mechanism, the stress-induced Hsp20 phosphorylation could safeguard cytoskeletal integrity and protection from injury during stress conditions. Conversely, the human phosphorylation impairing Hsp20-P20L mutation disrupts formation of the 14-3-3/Hsp20 protein complex, fails to displace CFL2 from 14-3-3 and leads to aberrant control of actin dynamics. Under stress conditions, this leads to cytoskeletal disruption and myofibrillar damage that is anticipated to affect cardiac contractility and contribute to cardiac pathology. Therapeutic approaches modulating Hsp20 phosphorylation may thus hold promise in combating heart disease.

## 4. Materials and Methods

### 4.1. Generation of Recombinant Proteins

The generation of the full length 14-3-3 gamma (amino acids 1–247) expression construct was performed by RT-PCR on human muscle cDNA using primer-1 (5′ GCGAAGATGGTGGACC 3′) and primer-2 (5′ CTTAATTGTTGCCTTCGCCG 3′). Deletion expression constructs of 14-3-3 were generated using primer-1 and primer-3 (5′ CTTTGCTCTCGTACTGGGTC 3′) (amino acids 1–120) or primer-4 (5′ ACCCAGTACGAGAGCAAAGTG 3′) and primer-2 (amino acids 115–247). PCR products were cloned in the EcoRI/SalI sites of the pMALc2x vector (New England Biolabs, Ipswich, MA, USA). The authenticity of all constructs was confirmed by sequence analysis (Macrogen Europe B.V., Amsterdam, The Netherlands). The expression constructs of GST-Hsp20 (amino acids 1–160) containing the wild-type or the Hsp20-P20L variant, as well as the different GST-CFL2 constructs containing full length and deletion fragments of CFL2, have been previously reported [16,53,57].

Protein expression of all of the above constructs was performed as previously described [58]. Recombinant proteins were purified by affinity chromatography on Glutathione SepharoseTM 4B Beads (GE Healthcare, Life Sciences, Buckinghamshire, UK) or amylose resin (New England Biolabs, Ipswich, MA, USA) and, where appropriate, fusion-peptides were eluted from the beads according to manufacturer’s instructions.

### 4.2. Pull down Assays

Pull down assays were performed as previously described [58,59]. Briefly, cardiac homogenates of mouse origin were prepared in 10 mM NaPO_4_ (pH 7.2), 2 mM EDTA, 10 mM NaN_3_, 120 mM NaCl and 1% NP-40, supplemented with protease inhibitors (Sigma-Aldrich, Munich, Germany). Equivalent amounts of recombinant MBP and MBP-14-3-3 recombinant proteins bound to amylose resin (New England Biolabs) were mixed with 0.5 mg of cardiac homogenates at 4 °C for 16 h. The beads were washed with 10 mM NaPO_4_ (pH 7.2), 10 mM NaN_3_, 120 mM NaCl, 0.1% (*v/v*) Tween-20 and were subsequently analyzed by Western blot using Hsp20, phospho-Hsp20 (Ser16) (AbCam, Cambridge, UK), CFL2 and phospho-CFL2 (Merck Millipore, Darmstadt, Germany) primary antibodies and peroxidase-conjugated goat anti-rabbit (GE Healthcare Life Sciences, Buckinghamshire, UK) secondary antibody. Immunoreactive bands were detected using Pierce ECL Plus reagents (ThermoFisher Scientific, Waltham, MA, USA).

### 4.3. Protein Phosphorylation

Protein phosphorylation of cardiac protein homogenates or GST-Hsp20 recombinant protein was performed in vitro using the cAMP-dependent protein kinase (PKA), catalytic subunit (New England Biolabs), as previously described [53]. Briefly, proteins were incubated with 1× PKA Reaction Buffer (50 mM Tris-HCl pH 7.5, 10 mM MgCl_2_), supplemented with 200 μM ATP (Sigma-Aldrich) and 1250 units of the PKA catalytic subunit. Samples were incubated at 30 °C for 1 h and subsequently used for pull down or blot overlay assays.

GST-CFL2 recombinant protein phosphorylation was performed as previously described [57]. In brief, GST-CFL2 was incubated with the cardiac protein extracts in 3.1 mM ATP, 3 mM NaF and 3 mM Na3VO4 at 25 °C for 30 min. The samples were washed three times with wash buffer (15 mM NaF, 2 mM Na_3_VO_4_, 50 mM Tris-HCl, pH 7.5, 150 mM NaCl, 0.05% (*v/v*) Tween-20). GST-CFL2 phosphorylation was confirmed by Western blotting using an anti-phospho-CFL2 antibody (Merck Millipore, MA, USA).

### 4.4. Immunoprecipitations

Immunoprecipitation experiments were performed in mouse cardiac extracts, as previously described [60,61]. Briefly, pre-clearing of the cardiac extracts was performed with protein-A/G agarose beads (Santa Cruz Biotechnology, Heidelberg, Germany) on a rotary wheel at 4 °C for 1 h. The pre-cleared protein extracts were then incubated overnight on a rotary wheel at 4 °C with mouse monoclonal 14-3-3 antibody (Santa Cruz Biotechnology) or mouse IgG control antibody (Jackson ImmunoResearch, Ely, UK) and protein-A/G agarose beads. Immunoprecipitates were collected by a 5 min spin at 2000 rpm, washed three times in PBS, and analyzed by Western blot analysis.

### 4.5. Blot Overlay Assays

Protein interactions were assessed in vitro by blot overlay assays, as previously described [16,53,57,60,62]. Briefly, purified GST, GST-Hsp20 and phosphorylated GST-Hsp20 recombinant proteins were separated by SDS-PAGE and transferred to nitrocellulose membranes. Following blocking, the membranes were incubated with MBP-14-3-3 fusion protein. The blots were probed with anti-MBP and the immunoreactive bands were visualized using ECL reagents. In another set of experiments, GST, GST-CFL2 and phosphorylated GST-CFL2 recombinant proteins were allowed to interact with MBP-14-3-3, as described above.

In a different set of experiments, mapping of the minimal binding domain of 14-3-3 to CFL2 was performed using equal amounts of GST, GST-CFL2 (amino acids 1–166), GST-CFL2 (amino acids 1–55), GST-CFL2 (amino acids 19–154) and GST-CFL2 (amino acids 105–166) proteins that were separated by SDS-PAGE and allowed to interact with MBP-14-3-3 (amino acids 1–247), as described above. Western blot analysis with an MBP antibody (New England Biolabs) determined the minimal region of CFL2 required for 14-3-3 binding.

In parallel, GST, GST-CFL2 and phosphorylated GST-Hsp20 recombinant proteins were allowed to interact with MBP-14-3-3 (amino acids 1–120) or MBP-14-3-3 (amino acids 115–247), as described above.

### 4.6. Actin Depolymerization Assay

The Actin Polymerization Biochem Kit (Cytoskeleton, Denver, CO, USA) was used to investigate actin dynamics in vitro on a PerkinElmer LS 55 Fluorescence spectrometer (PerkinElmer Ltd., Bucks, UK), as previously described [57,61]. The F-actin depolymerization was performed using GST-CFL2 and MBP-14-3-3 in the presence or absence of GST-Hsp20.

### 4.7. Cell Culture, Transfections and Immunofluorescence Studies

HEK 293 cells (ECACC, Salisbury, UK) were maintained in Dulbecco’s modified Eagle medium supplemented with 10% fetal bovine serum (ThermoFisher Scientific), as previously described [58,59]. The full-length Hsp20 mammalian expression constructs of wild-type or Hsp20-P20L were generated by PCR, using previously reported primers [53], while the full-length CFL2 construct was generated with primers 5′ATGGCTTCTGGAGTTACA 3′ and 5′ TGGCACTTGACTGTCATT 3′. PCR products were cloned in the pCMV-Tag3 (Stratagene, Amsterdam, The Netherlands) vector.

For immunofluorescence analysis, the myc-Hsp20 construct was transiently transfected in HEK 293 cells with Lipofectamine™ 2000 (ThermoFisher Scientific), according to the manufacturer’s instructions. Twenty-four hours after transfection, cells were fixed for 20 min at 25 °C with ice cold methanol, washed three times with phosphate-buffered saline (1× PBS) and permeabilized for 30 min at 25 °C in PBS containing 0.1% Triton X-100. The cells were then washed in PBS prior to incubation with blocking buffer (1× PBS, 1 mg/mL BSA and 10 mM NaN_3_) for 1 h at 25 °C. Hsp20 (AbCam) or actin (Merck Millipore) primary antibodies were diluted in blocking buffer and applied to the cells for 1 h at 25 °C. Samples were washed again with PBS and counterstained for 1 h at 25 °C with the appropriate secondary antibody (anti-rabbit Alexa Fluor 488, or anti-Alexa Fluor mouse 568) (Invitrogen) diluted in blocking buffer. Following further washes with PBS, samples were mounted with Vectashield medium containing DAPI (Vector Laboratories, Burlingame, CA, USA) and analyzed on a Leica confocal laser scanning microscope (Leica TCS SP5 on a DMI6000 Inverted Microscope, with the acquisition software program LAS-AF). Localization analysis for Hsp20 and actin was performed using Colocalization Threshold plugin of ImageJ.

### 4.8. PCR Mutagenesis

For the generation of Hsp20-S16A and Hsp20-S16D constructs, PCR mutagenesis was performed on WT Hsp20 myc-tagged or GST-tagged plasmids, as previously described [63]. Specifically, for Hsp20-S16A constructs, primers 5′ TTGGCTGCGCCGCGCCGCCGCCCCGTTGCCCGGACTTTCGG 3′ and 5′ CCGAAAGTCCGGGCAACGGGGCGGCGGCGCGGCGCAGCCAA 3′ were used, while primers 5′ TTGGCTGCGCCGCGCCGACGCCCCGTTGCCCGGACTTTCGG 3′ and 5′ CCGAAAGTCCGGGCAACGGGGCGTCGGCGCGGCGCAGCCAA 3′ were used for the generation of Hsp20-S16D constructs.

### 4.9. Cell Fractionation

Cell fractionation experiments were performed as previously described [5]. In brief, transfected HEK 293 cells were harvested and resuspended in ice-cold lysis buffer containing 10 mM Tris pH 7.5, 10 mM NaCl, 5 mM MgCl_2_, 1 mM phenylmethanesulfonyl fluoride and 0.5% Triton X-100, supplemented with protease inhibitors. Samples were centrifuged for 5 min at 2000 rpm and 4 °C, and the supernatant was used as the detergent-soluble fraction. The pellet was washed with the same buffer and then used as the detergent-insoluble fraction. For the isoproterenol treatment, cells were incubated for 5 min with 1 μM isoproterenol (ISO) at 37 °C (Sigma-Aldrich) and then harvested for fractionation experiments, as described above.

### 4.10. Protein Binding Competition Experiments

The competition of Hsp20 and CFL2 for 14-3-3 binding was examined by pull down assays. In particular, HEK 293 cells were transfected with the full length myc-CFL2 construct and after 24 h the cells were lysed in 50 mM Tris-HCl, pH 8.0, 150 mM NaCl and 1% NP-40, supplemented with protease inhibitors (Sigma-Aldrich). Pull down assays were performed using myc-CFL2 HEK 293 cell lysates that were incubated with MBP-14-3-3 recombinant protein bound to amylose resin, in the presence of phosphorylated or non-phosphorylated GST-Hsp20 eluted protein. Protein binding to MBP-14-3-3 was determined by Western blot analysis using myc (Sigma-Aldrich) or phospho-Hsp20 (AbCam) antibodies.

## Supplementary Materials

The following are available online at https://www.mdpi.com/1422-0067/21/24/9572/s1.

## Abbreviations

Hsp20	Heat shock protein 20
CFL2	cofilin-2
DCM	dilated cardiomyopathy
Ser16	Ser16
PKA	cAMP-dependent protein kinase
ISO	isoproterenol
