# Supplementary material for: The Cardioprotective PKA-Mediated Hsp20 Phosphorylation Modulates Protein Associations Regulating Cytoskeletal Dynamics
Source: Int J Mol Sci. 2020 Dec 16;21(24):9572. doi: 10.3390/ijms21249572 (PMC7765622; doi:10.3390/ijms21249572)
Supplement: Supplementary file 1 [file ijms-21-09572-s001.pdf]

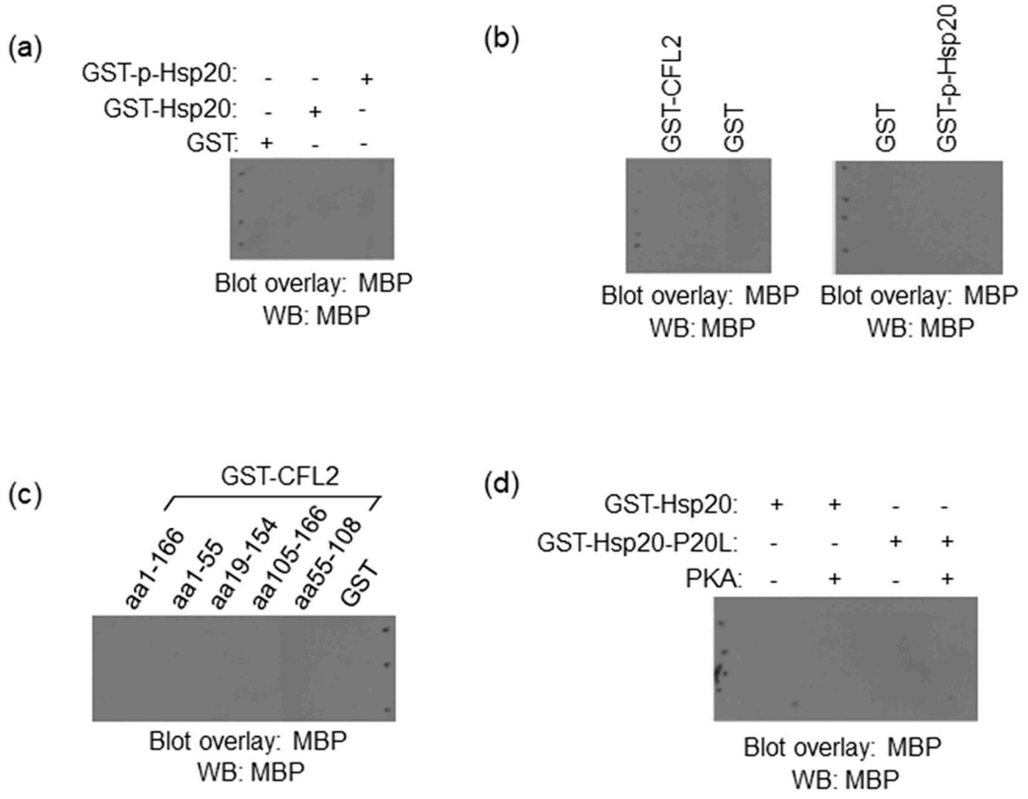

Supplementary Figure 1: No unspecific binding to MBP was observed in control blot overlay experiments. (a-b) Phosphorylated or non-phosphorylated GST-Hsp20, (b-c) GST-CFL2 full length or deletion constructs and (d) phosphorylated or non-phosphorylated GST-Hsp20-P20L were overlaid with MBP recombinant protein. Lack of MBP binding confirmed the specificity of GST-pHsp20/MBP-14-3-3, GST-CFL2/MBP-14-4-3 and GST-pHsp20-P20L/MBP-14-3-3 interactions.
